# Longitudinal Symptom Analysis of COVID-19 Survivors and Post-COVID Syndrome Patients

**DOI:** 10.3390/biomedicines13061334

**Published:** 2025-05-29

**Authors:** Eduarda Martins de Faria, Cíntia Moraes de Sá Sousa, Caroline de Oliveira Ribeiro, Márcio Neves Bóia, Agnaldo José Lopes, Pedro Lopes de Melo

**Affiliations:** 1 Biomedical Instrumentation Laboratory, Institute of Biology and Faculty of Engineering, State University of Rio de Janeiro, Rio de Janeiro 20550-013, Brazil; martiinseduarda09@gmail.com (E.M.d.F.); cintiamdesa@gmail.com (C.M.d.S.S.); caroline.oliveira@live.com (C.d.O.R.); 2 Infectious Parasitic Diseases Sector, Faculty of Medical Sciences, State University of Rio de Janeiro, Rio de Janeiro 20551-030, Brazil; marcioboiam@gmail.com; 3 Pulmonary Function Laboratory, Pedro Ernesto University Hospital, Faculty of Medical Sciences, State University of Rio de Janeiro, Rio de Janeiro 20551-030, Brazil; agnaldolopes.uerj@gmail.com; 4 Laboratory of Clinical and Experimental Research in Vascular Biology, Biomedical Center, State University of Rio de Janeiro, Rio de Janeiro 20551-030, Brazil

**Keywords:** COVID-19, SARS-CoV-2, post-COVID, remote patient monitoring, fatigue

## Abstract

**Background/Objectives:** The present study aimed to analyze changes in symptom intensity during the recovery period of COVID-19 survivors and patients with post-COVID syndrome. **Methods:** Initially, we described a new remote patient monitoring system to track the intensity of specific symptoms in individuals’ home environments. Remote patient monitoring (RPM) was implemented over 15 days in a cohort of 133 individuals aged 20 to 78 years, divided into four groups: mild (MG, n = 40), Hospital Discharge Without Invasive Mechanical Ventilation (WIMV, n = 40), Hospital Discharge With Invasive Mechanical Ventilation (IMV, n = 13), and reinfected (RG, n = 40). **Results:** The most prevalent symptoms reported across all groups, based on average intensity, were shortness of breath, fatigue, cough, headache, and body pain. The WIMV group exhibited the highest average intensities in six symptoms (*p* < 0.01), while the IMV group reported the highest averages in four symptoms (*p* < 0.05). Fatigue was the symptom with the highest overall intensity, followed by memory lapses. The hospitalized groups demonstrated the highest intensities and most persistent symptoms (*p* < 0.05). Blood pressure was significantly higher in the MG group compared to the RG group (*p* < 0.0001), although all values remained within the normal range. **Conclusions:** These results provide novel insights, revealing distinct differences in the symptom profiles among the studied groups. These findings hold significant implications for developing more personalized care strategies and informing future pandemic preparedness and response efforts.

## 1. Introduction

The Coronavirus Disease 2019 (COVID-19), caused by the novel severe acute respiratory syndrome coronavirus 2 (SARS-CoV-2), led to an unprecedented global health crisis. The primary symptoms manifested are respiratory, and other organs can also be affected [1]. Clinical manifestations can include fever or chills, cough, shortness of breath or difficulty breathing, fatigue, muscle or body pain, headache, loss of smell and/or taste, sore throat, nasal congestion, nausea, vomiting, and diarrhea [2].

Post-COVID-19 syndrome, frequently referred to as “Long COVID,” emerged as a significant concern following the initial peak of the pandemic. While the acute phase of COVID-19 often resolves within weeks, many individuals experience persistent or new symptoms lasting for months or even longer. The variety of symptoms and sequelae that may appear after recovery from SARS-CoV-2 infection are diverse and can significantly affect the quality of life of affected individuals [3]. Previous research suggests that the incidence of post-COVID is substantial, with estimates ranging from 10% to 30% of COVID-19 cases, impacting millions worldwide [4]. Post-COVID stands out as a fluctuating and episodic condition, with its severity potentially varying over time in the same individual [5,6]. Therefore, it requires ongoing care and monitoring of symptom improvement, which can be assessed using a scale from 0 to 10 [4].

Considering the large number of symptomatic COVID-19 survivors, there has been an expansion in the use of virtual healthcare, employing remote patient monitoring (RPM) as a mechanism to assess and follow up with patients at home, collecting health data such as symptom intensity and vital signs, which are transmitted to a healthcare provider [7]. This adaptation helped minimize the risk of exposure to healthcare professionals by enabling the management of patients with mild or stable symptoms and contributing to the reduction in community spread. Additionally, proper education on home care for exposed or infected individuals was essential in this process, supporting efforts to contain the virus [8].

The RPM can be used in various circumstances, such as in patients with active COVID-19 infection or those recently discharged from the hospital who require follow-up to identify potential clinical deterioration. It also supports patients experiencing a slower or incomplete recovery or those whose condition worsens over time, providing greater safety for these individuals [7,9]. This method may help in understanding the underlying mechanisms, risk factors, and long-term consequences of COVID, which is crucial for developing effective management strategies and mitigating its impact on individuals and healthcare systems.

This paper will explore the symptomatic changes during the recovery process of COVID-19 survivors and patients with post-COVID syndrome using RPM, contributing to the growing body of knowledge surrounding COVID-19 and informing future pandemic preparedness and response efforts.

## 2. Materials and Methods

### 2.1. Study Design and Ethics

This is an analytical, descriptive longitudinal study on the home monitoring of COVID-19 survivors and post-COVID syndrome patients. The study adheres to the Helsinki Declaration and Resolution No. 466/2012 guidelines. It was approved by the Research Ethics Committee of Pedro Ernesto University Hospital (CEP/HUPE). Participants were previously informed about the type of research and its objectives. After receiving information about the study, participation was consented to by signing the informed consent form.

### 2.2. Remote Patient Monitoring System

**Figure 1:**
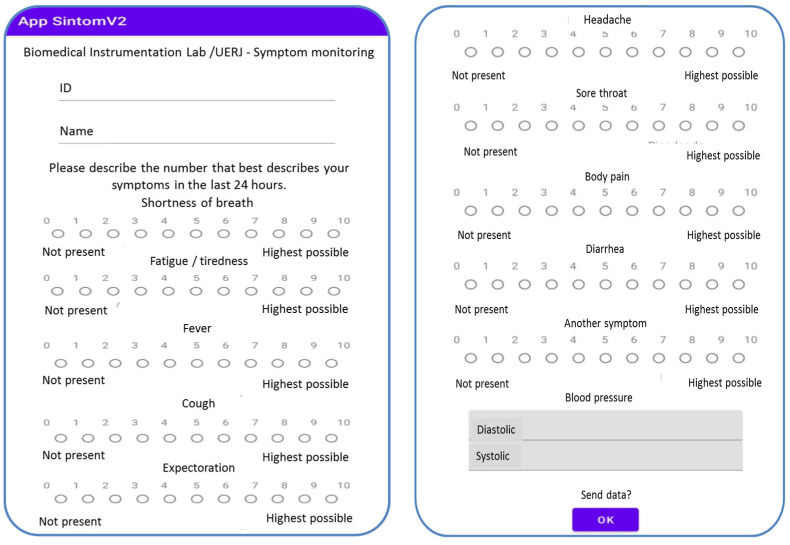
SintomV2 mobile application screens. Abbreviations: ID—identity number received at the beginning of the study.

Initially, the volunteers were instructed to download the SintomV2 mobile application (Figure 1), which was developed in our laboratory specifically for this study. The application contains a questionnaire with symptoms related to COVID-19 and a field for inputting blood pressure (BP). The blood pressure monitor (OMRON Control+ HEM-7122 Arm Blood Pressure Monitor—Jundiaí, SP, Brazil) was provided free of charge to the volunteers.

The used scale was originally developed by Bruera and colleagues with a focus on palliative care [10]. We adapted this scale for COVID-19 studies based on the symptoms described by Wan and colleagues [11]. The monitored symptoms include shortness of breath, fatigue/tiredness, fever/chills, cough, sputum, headache, sore throat, body pain, diarrhea, and other symptoms, with “other” considered any additional symptom the volunteer was experiencing that was not listed in the questionnaire. Below each symptom is a numerical rating scale from 0 to 10, where 0 indicates the absence of the symptom and 10 represents the worst possible sensation. Following previous studies, the intensity of symptoms was evaluated once per day for 15 days [7,8,12,13,14,15]. These values were automatically uploaded via the internet to a monitoring spreadsheet on Google Drive, allowing real-time access.

Blood pressure was subsequently included in the analysis after observing the decompensation of this variable in patients presenting post-COVID symptoms who visited the laboratory for pulmonary function tests. As BP monitoring was incorporated during the project’s development, not all volunteers measured this variable.

**Figure 2:**
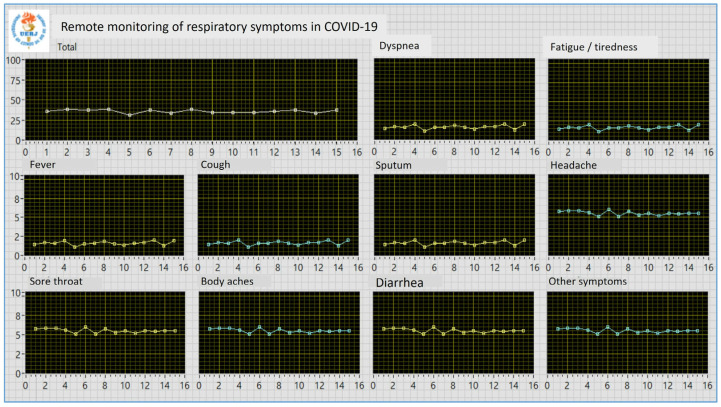
User-friendly front panel developed for the monitoring environment. The program automatically downloads the patient’s measurements and updates the graphs.

A dedicated software was also developed in our laboratory for the monitoring environment. It was developed in the LABVIEW 2020 environment (National Instruments, Austin, TX, USA), providing a user-friendly front panel. This panel is shown in Figure 2.

The system allows real-time updating of all graphics describing the behavior of the studied symptoms, including the sum of all symptoms. This assessment determined the need to advise the patient to seek medical care from their trusted healthcare provider. We contacted the individuals to check their health status when very high intensities or abrupt changes were detected.

### 2.3. Subjects

All studied individuals were over 18 years old, had a history of a positive COVID-19 test through RT-PCR, and had recovered from the infection. Post-COVID-19 syndrome was defined according to [16].

A total of 133 individuals were monitored from April 2021 to December 2023 and distributed into four groups:

(1) Forty individuals in the Mild Group (MG), which consisted of volunteers who recovered at home without the need for hospitalization;
(2) Forty volunteers in the hospital discharge group, without the use of Invasive Mechanical Ventilation (WIMV). This group consisted of volunteers who required hospitalization, underwent oxygen therapy and/or non-invasive ventilation (NIV), but did not need intubation;
(3) Thirteen in the hospital discharge group with IMV (IMV). This group consisted of volunteers who required hospitalization and orotracheal intubation;
(4) Forty patients in the Reinfected Group (RG), which consisted of volunteers who experienced at least one reinfection with SARS-CoV-2, regardless of prior infection history. The essential criterion for inclusion in this group was at least one confirmed reinfection, which occurred at least 15 days after the first infection. This includes both individuals who recovered at home and those who required hospitalization.

### 2.4. Protocol

Initially, data on age, weight (kg), and height (cm) were collected, allowing the calculation of body mass index (BMI). In addition to this information, volunteers also provided details about the development of the disease, medications used for COVID-19 treatment, and other health conditions. Volunteers were provided with email and phone contacts for inquiries.

### 2.5. Statistics

The estimative of the number of volunteers was performed using Medcalc version 12.3 and based on a pilot study including 16 individual in the control and WIMV groups. Type 1 errors of 10% and type 2 errors of 20% were assumed to be acceptable, resulting in a value of 40 individuals per group.

The results were presented as mean ± standard deviation (SD), either longitudinally or as percentage descriptions. Initially, the distribution characteristics of the sample were evaluated using the Shapiro–Wilk test. Since the data were not normally distributed, non-parametric analyses were performed using the Mann–Whitney test. Differences with *p* ≤ 0.05 were considered statistically significant. The analyses and graphs were performed using Origin^®^ 8.0 (Microcal Software Inc., Northampton, MA, USA).

## 3. Results

The anthropometric characteristics of the studied groups are described in Table 1. Regarding age, the WIMV group showed a significant increase when compared to the MG and RG groups. As for weight, both the WIMV and IMV groups showed a significant increase compared to the MG and RG groups. Regarding BMI, there was a significant decrease between the WIMV and IMV groups and a significant increase between the WIMV and RG groups. Additionally, the WIMV group significantly increased compared to the MG and RG groups. No significant differences in height were observed between the groups.

The clinical characteristics of the studied patients are described in Table 2. Few volunteers were smokers (mean 3.8%). However, the WIMV group had the highest percentage of former smokers (32.5%). The majority of the volunteers were obese (mean 39.75%) and had hypertension (mean 34.75%) as the most common comorbidity. Most participants (mean 39.7%) used Azithromycin as a treatment for COVID-19, compared to Hydroxychloroquine and Ivermectin (mean 15.78).

**Figure 3:**
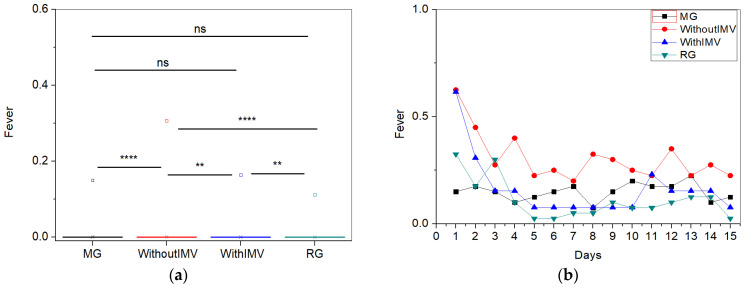
Comparisons between the mean values of the studied groups (**a**) and the longitudinal monitoring of the fever symptom (**b**). Abbreviations: MG—Mild Group; WIMV—Post-Discharge Group Without Invasive Mechanical Ventilation; IMV—Post-Discharge Group With Invasive Mechanical Ventilation; RG—Reinfected Group; IMV—Invasive Mechanical Ventilation. Notes: ns—not significant; ** *p* < 0.01; **** *p* < 0.0001.

The comparisons between the mean values and the longitudinal follow-up of the fever symptom are shown in Figure 3a and Figure 3b, respectively. The WIMV group presented the highest mean intensity for fever (0.30 ± 0.80) (Figure 3a). As observed in Figure 3b, patients in the MG group exhibited relatively low and stable fever intensity, with minimal variations over the 15 days. The values in the WIMV group started with a slightly higher fever sensation in the initial days, which decreased after the fourth day but remained relatively higher than the other groups. Those in the WIMV group showed reduced symptoms in the first days, with minor fluctuations over time. The RG group also demonstrated variability in intensity during the initial days but then stabilized similarly to the other groups. Regardless of the group, the fever sensation was higher during the first days of monitoring, decreasing and stabilizing over time.

**Figure 4:**
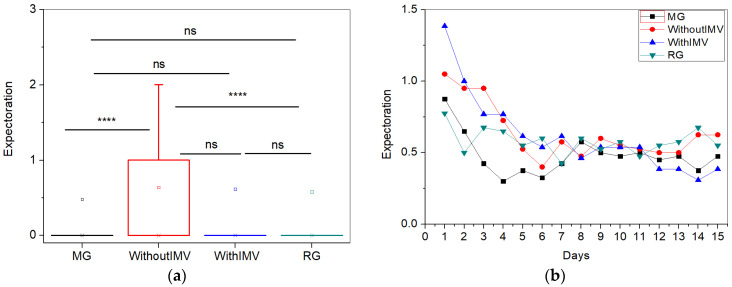
Comparisons between the mean values of the studied groups (**a**) and the longitudinal monitoring of the expectoration symptom (**b**). Notes: ns—not significant; **** *p* < 0.0001.

For the symptom of expectoration, the comparisons between the mean values are shown in Figure 4a, where the WIMV group exhibited the highest mean intensity (0.63 ± 1.22). The longitudinal follow-up of the symptom (Figure 4b) shows that at the beginning of the monitoring, all groups showed relatively higher levels of expectoration, which gradually decreased over the days. Patients in the MG and RG exhibited the lowest intensities. After the sixth day, all groups stabilized sputum levels with minor fluctuations. Expectation tends to decrease over time in all patient groups; however, the regularity of this reduction varies between the groups.

**Figure 5:**
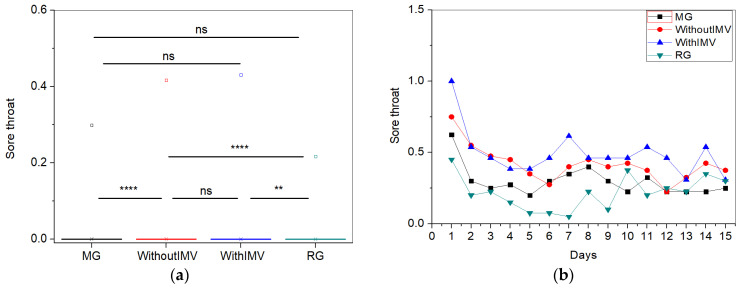
Comparisons between the mean values of the studied groups (**a**) and the longitudinal monitoring of the sore throat symptom (**b**). Notes: ns—not significant; ** *p* < 0.01, **** *p* < 0.0001.

For the sore throat symptom (Figure 5a), the WIMV group exhibited the highest mean intensity (0.43 ± 1.20). Regarding the longitudinal follow-up (Figure 5b), all groups began with varied levels of sore throat, with patients in the WIMV group presenting the highest initial levels. Over the 15 days, sore throat levels decreased in all groups during the first two days. Throughout the monitoring period, sore throat levels fluctuated, with the WIMV group showing the most significant variations. The MG and RG maintained relatively lower intensities after the initial days, while patients in the WIMV and IMV groups continued to experience fluctuations. Sore throat tends to decrease rapidly for all groups in the first few days, stabilizing at low. However, persistent and oscillatory levels were observed, particularly in patients from the WIMV group. This indicates that these patients may have a more complicated and prolonged recovery from this symptom.

**Figure 6:**
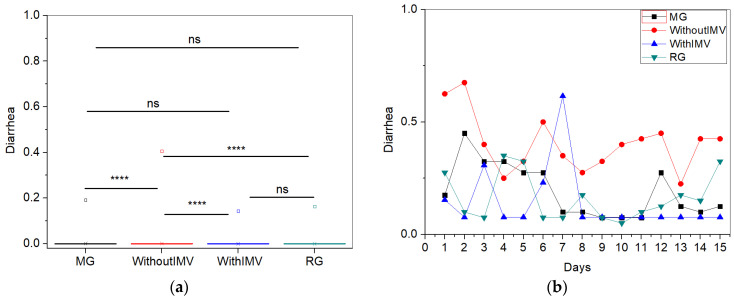
Comparisons between the mean values of the studied groups (**a**) and the longitudinal monitoring of the symptoms of diarrhea (**b**). Notes: ns—not significant; **** *p* < 0.0001.

For the symptom of diarrhea, the WIMV group exhibited the highest mean intensity (0.40 ± 1.06) (Figure 6a). Regarding the symptom follow-up (Figure 6b), the WIMV group showed the highest symptom levels at the beginning of monitoring, while the other groups began with lower levels. Over the days, there is significant variability, especially in the WIMV patients, who maintain higher and more unstable levels. The MG and RG displayed less intense occurrences, while the WIMV group experienced occasional peaks, such as on the third and seventh days. Diarrhea showed a more consistent presence in the WIMV group, while the other groups, particularly MG and RG, had lower symptom levels over time.

**Figure 7:**
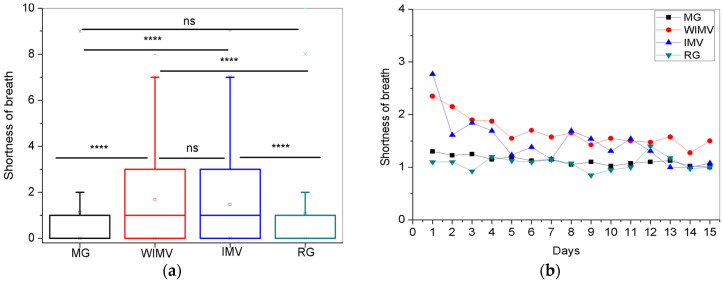
Comparisons between the mean values of the studied groups (**a**) and the longitudinal monitoring of the shortness of breath symptom (**b**). Notes: ns—not significant; **** *p* < 0.0001.

In Figure 7a, the comparisons between the mean values of the shortness of breath symptom show that the WIMV group presented the highest mean intensity (1.67 ± 2.01). Figure 7b shows the longitudinal monitoring of shortness of breath. Throughout the monitoring period, patients in both the WIMV and IMV groups exhibited the highest intensities, mainly in the early days of monitoring. The MG and RG maintained consistently low and stable levels. Over the days of monitoring, there was a general tendency for the shortness of breath symptoms to decrease across all groups.

**Figure 8:**
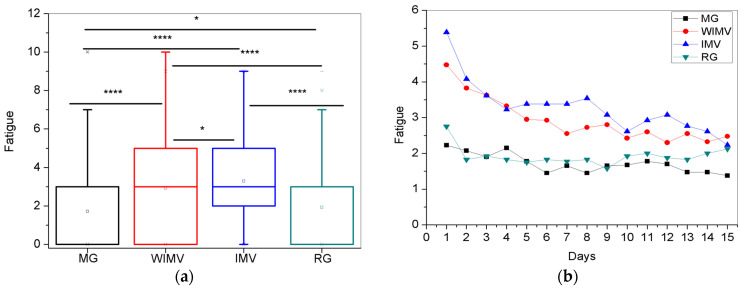
Comparisons between the mean values of the studied groups (**a**) and the longitudinal monitoring of the fatigue symptom (**b**). Notes: ns—not significant; * *p* < 0.05, **** *p* < 0.0001.

For the symptom of fatigue, the comparison between the groups is shown in Figure 8a, and the longitudinal follow-up is presented in Figure 8b. In Figure 8a, the WIMV group presented the highest mean intensity for this symptom (3.28 ± 2.31). Figure 8b shows that initially, individuals in the IMV group showed the highest levels of fatigue, closely followed by the WIMV group. The MG and RG exhibited lower symptom intensities from the beginning of the monitoring and remained relatively constant and stable throughout this period. Over time, a gradual decrease in fatigue was observed in the IMV and WIMV groups, though they continued to display higher intensities than the MG and RG groups.

**Figure 9:**
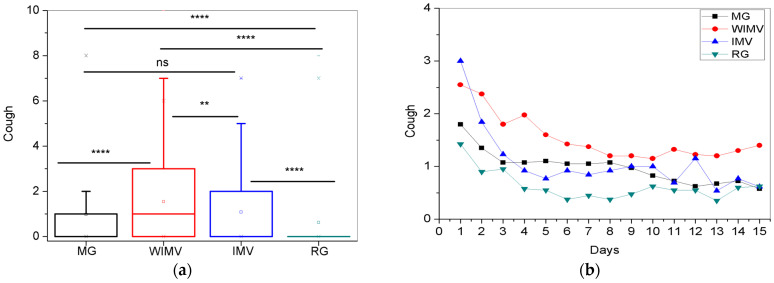
Comparisons between the mean values of the studied groups (**a**) and the longitudinal monitoring of the cough symptom (**b**). Abbreviations: MG—Mild Group; WIMV—Post-Discharge Group Without Invasive Mechanical Ventilation; IMV—Post-Discharge Group With Invasive Mechanical Ventilation; RG—Reinfected Group; IMV—Invasive Mechanical Ventilation. Notes: ns—not significant; ** *p* <0.01; **** *p* <0.0001.

The comparisons between the mean values and the longitudinal follow-up of the cough symptom are shown in Figure 8a and 8b, respectively. Figure 9a shows that the WIMV group exhibited the highest mean intensity (1.54 ± 1.90). Analyzing Figure 9b, the IMV group showed the highest intensity of coughing symptoms on the first monitoring day, followed by the WIMV group. In contrast, the MG and RG groups consistently displayed lower intensities. However, despite the IMV group presenting the highest intensity on the first day, it can be seen that by the second day, there was a decrease in the intensity of the symptom, with the WIMV group presenting the highest intensity for the remainder of the monitoring period. Over time, all groups showed a tendency for the cough symptoms to decrease.

**Figure 10:**
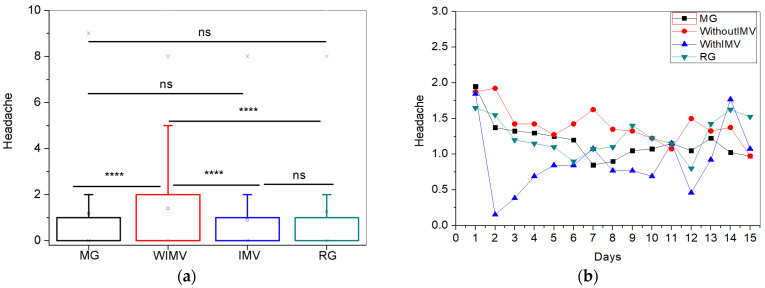
Comparisons between the mean values of the studied groups (**a**) and the longitudinal monitoring of the headache symptom (**b**). Abbreviations: MG—Mild Group; WIMV—Post-Discharge Group Without Invasive Mechanical Ventilation; IMV—Post-Discharge Group With Invasive Mechanical Ventilation; RG—Reinfected Group; IMV—Invasive Mechanical Ventilation. Notes: ns—not significant; **** *p* <0.0001.

For the headache symptom, the WIMV group exhibited the highest mean intensity (1.40 ± 2.00) (Figure 10a). Figure 10b illustrates the longitudinal monitoring of this symptom. Initially, all groups showed similar levels of headache intensity, with a marked decrease on the second day for the IMV group, followed by significant fluctuations throughout the remainder of the monitoring period. The WIMV group maintained higher headache intensity over time, while the MG and RG displayed less pronounced variations. Despite the fluctuations, the headache symptoms decreased over time in all groups. Patients in the WIMV group tended to have higher headache levels than the other groups, and the IMV group displayed more pronounced fluctuations than the other groups.

**Figure 11:**
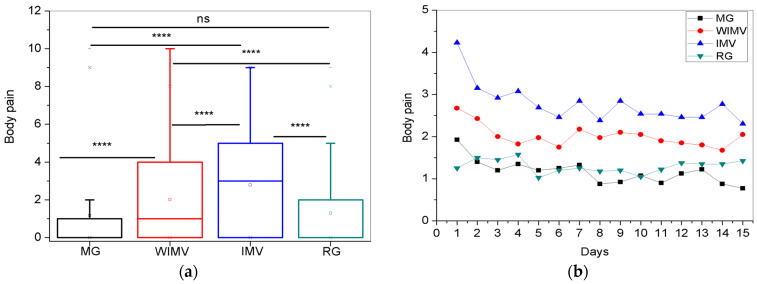
Comparisons between the mean values of the studied groups (**a**) and the longitudinal monitoring of body pain symptoms (**b**). Abbreviations: MG—Mild Group; WIMV—Post-Discharge Group Without Invasive Mechanical Ventilation; IMV—Post-Discharge Group With Invasive Mechanical Ventilation; RG—Reinfected Group; IMV—Invasive Mechanical Ventilation. Notes: ns—not significant; **** *p* <0.0001.

For the body pain symptoms, a comparison between the groups is shown in Figure 11a. The IMV group exhibited the highest mean intensity (2.77 ± 2.43). The longitudinal monitoring is represented in Figure 11b. The IMV group presented the highest levels of pain. Although a gradual decrease was observed, it was the highest level over the monitoring period. The WIMV group also showed higher levels than the MG and RG groups but with less pronounced fluctuations, indicating a stabilization in the intensity of this symptom. The MG and RG groups exhibited the lowest intensities, remaining stable and symmetrical throughout the monitoring period.

**Figure 12:**
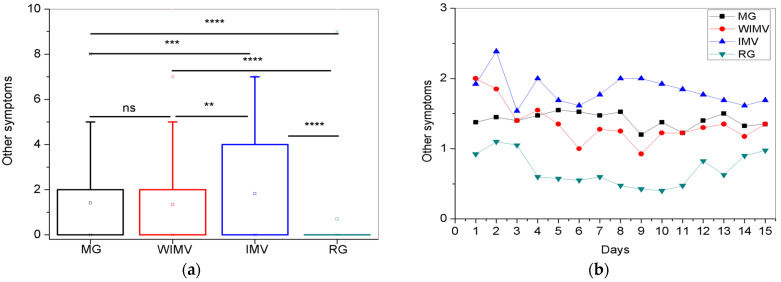
Comparisons between the mean values of the studied groups (**a**) and the longitudinal follow-up of other symptoms (**b**). Abbreviations: MG—Mild Group; WIMV—Post-Discharge Group Without Invasive Mechanical Ventilation; IMV—Post-Discharge Group With Invasive Mechanical Ventilation; RG—Reinfected Group; IMV—Invasive Mechanical Ventilation. Notes: ns—not significant; ** *p* < 0.01; *** *p* < 0.001; **** *p* < 0.0001.

Regarding the other symptoms, the comparison between the groups is shown in Figure 12a, and the longitudinal monitoring is presented in Figure 12b. As seen in Figure 12a, the IMV group exhibited the highest mean intensity. According to Figure 12b, the IMV group showed the highest and most persistent intensities throughout the monitoring period. However, there was a general trend of a gradual decrease in intensity over time. The WIMV group demonstrated a gradual reduction in the symptom intensity. The MG group exhibited some fluctuations, with increased intensity followed by gradual decreases, generally remaining stable with few variations. The RG group showed the lowest intensity levels, with a decrease and stabilization between days 4 and 11, followed by fluctuations until the end of the monitoring period.

To improve the interpretation of the results, Table 3 resumes the mean and standard deviation of the symptoms monitored in this study. Fever, expectoration, sore throat, and diarrhea exhibited the lowest intensity values. In contrast, the symptoms of shortness of breath, fatigue, cough, headache, body pain, and others showed the highest values. The WIMV group presented the highest mean intensity for most of the symptoms. The MG and RG groups did not show higher intensities for the symptoms studied.

Table 4 describes the other symptoms reported by the volunteers included in the study, where memory lapse was the most prevalent symptom (10.5%), followed by tachycardia (5.26%) and tingling in the limbs (3.0%). No other symptoms were reported by 56.4% of the volunteers.

**Figure 13:**
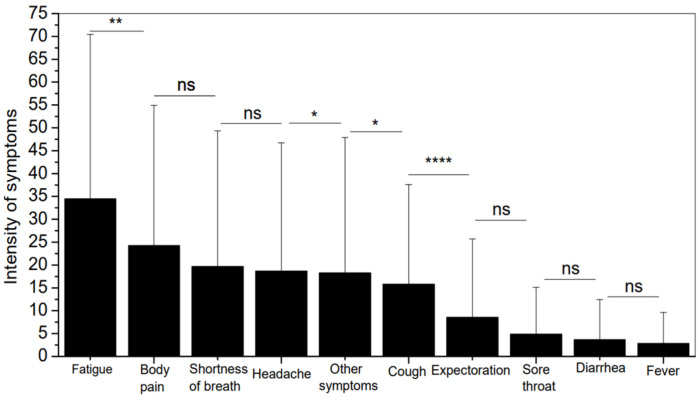
Mean level and SD of each symptom over the monitoring period for all studied individuals. Notes: ns—not significant; * *p* <0.05; ** *p* <0.01; **** *p* <0.0001.

Figure 13 presents each symptom’s mean level and standard deviation (SD) throughout the monitoring period for all the individuals studied. Fatigue was the symptom with the highest intensity, followed by body pain and shortness of breath. The interested reader may find the exact values of the observed mean and standard deviation of the intensity of all symptoms in the online appendix (Table A1).

**Figure 14:**
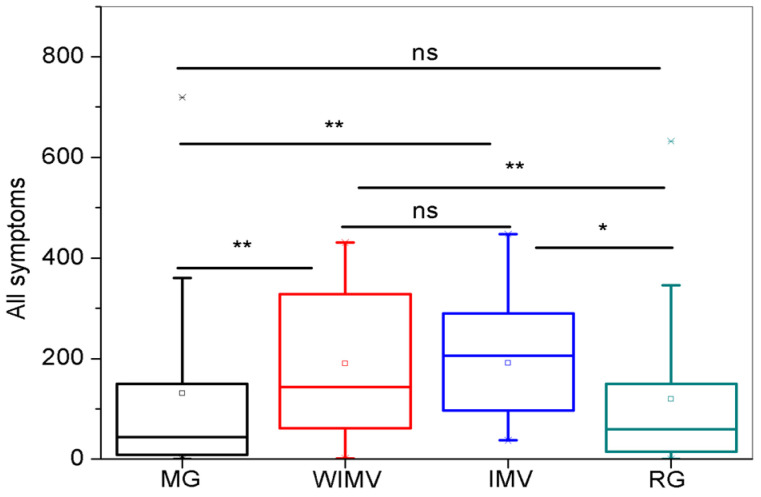
Comparisons between the groups regarding the intensity of all studied symptoms. Abbreviations: MG—Mild Group; WIMV—Post-Discharge Group Without Invasive Mechanical Ventilation; IMV—Post-Discharge Group With Invasive Mechanical Ventilation; RG—Reinfected Group; IMV—Invasive Mechanical Ventilation. Notes: ns—not significant; * *p* < 0.05; ** *p* < 0.01.

Figure 14 shows a comparative analysis of all symptoms among the studied groups. The WIMV and IMV groups presented significantly higher values than the MG and RG. The comparisons among the MG and RG groups and the WIMV and IMV groups did not show statistical significance. The interested reader may find these exact values in the online appendix (Table A2).

**Figure 15:**
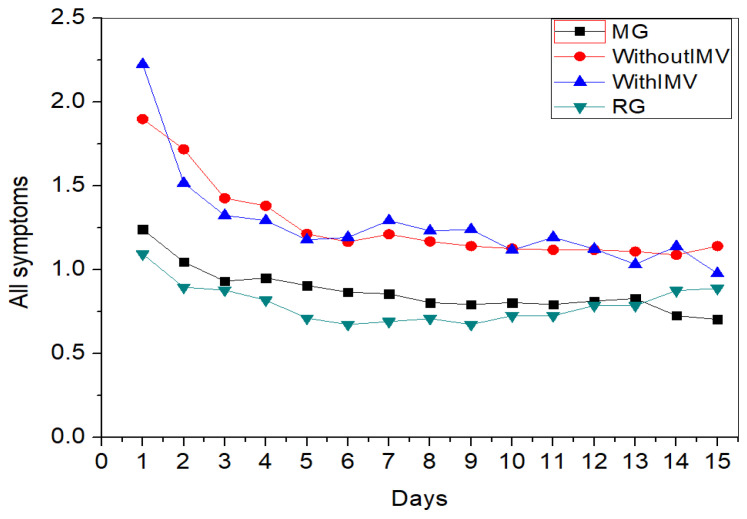
Longitudinal follow-up of monitoring between groups in the intensity of all studied symptoms. Abbreviations: MG—Mild Group; WIMV—Post-Discharge Group Without Invasive Mechanical Ventilation; IMV—Post-Discharge Group With Invasive Mechanical Ventilation; RG—Reinfected Group.

Figure 15 presents the longitudinal monitoring of the sum of the intensity of symptoms. It can be observed that the IMV group starts with the highest intensity but is still very similar to the WIMV group. Throughout the monitoring period, the intensities remained similar between the groups, gradually decreasing. The MG and RG groups exhibited lower and similar intensities, remaining stable with minimal fluctuations.

**Figure 16:**
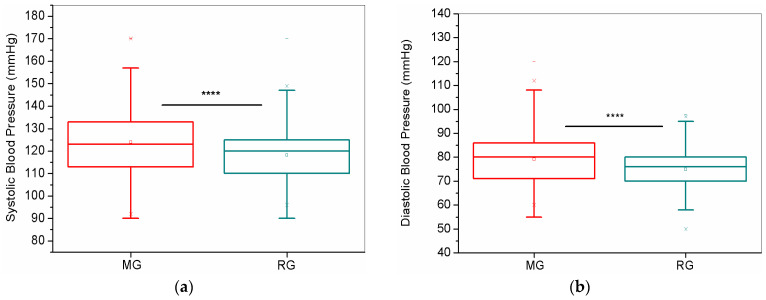
Comparisons of SBP (**a**) and DBP (**b**) between the MG and RG. Abbreviations: MG—Mild Group; RG—Reinfected Group. Notes: **** *p* < 0.0001.

Figure 16 shows the Systolic Blood Pressure (SBP) and Diastolic Blood Pressure (DBP) between the MG and RG groups. The MG group presented the highest mean for Systolic Blood Pressure (SBP) (*p* < 0.0001) and Diastolic Blood Pressure (DBP) (*p* < 0.0001).

Additionally, 31.5% of the patients experienced extremely intense symptoms or elevated blood pressure and were advised to seek medical attention during the monitoring period. Among these individuals, 38.0% followed the recommendation and sought care, while 62.0% did not.

## 4. Discussion

This study describes the implementation and evaluation of an RPM method used to assess the recovery of both COVID-19 survivors and patients diagnosed with post-COVID syndrome. Our findings indicate that this RPM approach facilitates the early detection of complications. Participants reported positive experiences with the tool, expressing gratitude and a sense of enhanced care during their recovery. Furthermore, the study revealed distinct differences in the reported symptoms between the groups. These findings are significant as they contribute to more personalized care strategies and inform future pandemic preparedness and response initiatives.

Table 1 presents the anthropometric characteristics of the studied individuals. Regarding age, the WIMV and IMV groups had the highest averages, with the IMV group also showing the highest body weight and BMI. Differences among basic parameters (age, BMI, etc.) may seem strange in the first moment. However, in the present study, these differences are closely related to the outcomes of the disease under study. In close agreement with our results, previous studies, including those by Abate et al. [17] and Soeroto and colleagues [18], reported that age is associated with unfavorable outcomes in COVID-19 patients. Qadar and collaborators [19] highlighted an association between obesity and severe COVID-19. The authors pointed out that obesity is highly prevalent among patients requiring Intensive Care Unit (ICU) admission and that patients with adverse outcomes, such as hospitalization, use of IMV, or death, frequently had a high BMI. They suggested that the association between obesity and adverse outcomes is influenced by age.

The clinical characteristics of the volunteers are described in Table 2. It can be observed that the majority of the volunteers were obese (35.3%), had hypertension (29.3%) as the most common comorbidity, and used Azithromycin (36.8%) as a treatment for COVID-19. Few volunteers were active smokers (3.0%). However, the majority of former smokers were in the WIMV group (32.5%). Regarding smoking, two meta-analyses indicated that smoking increases the risks of adverse outcomes in patients with active SARS-CoV-2 infection [20,21]. Barthélémy and colleagues [22] observed that smoking is associated with the risk of developing post-COVID syndrome. Additionally, Hossain et al. [23] reported that smoking was a predictor of long-term COVID-19, directly influencing the longer duration of symptoms.

It can be observed from Table 3 that the symptoms of fever (Figure 3a), expectoration (Figure 4a), sore throat (Figure 5a), and diarrhea (Figure 6a) presented the lowest intensity values. Regarding fever (Figure 3a), the group with the highest intensity of fever was the WIMV group (0.30 ± 0.80). Daanen et al. [24] and Ahmad and colleagues [25] discuss that, during a viral infection, the immune system is activated, raising the body temperature to create an unfavorable environment for the virus, resulting in fever. Therefore, fever is not expected in the absence of an active infection. This mechanism may explain the low fever intensities found in all groups, which are associated with the resolution of the infection.

Regarding the longitudinal aspect presented for fever in Figure 3b, its intensity is higher in the initial days of monitoring, decreasing and stabilizing over time. In most individuals, the fever present in the early stage of the disease resolves over time [4]. However, the persistence of this symptom may be attributed to a persistent inflammatory response. As discussed earlier, SARS-CoV-2 infection can trigger inflammation that persists even after the resolution of the acute infection. This suggests that the immune system may continue to produce inflammatory cytokines, resulting in the maintenance of fever. It is well known that several pro-inflammatory cytokines have endogenous pyrogenic effects on the system [26].

Another factor that could influence the persistence of fever is the individual’s immune dysfunction, meaning the immune system does not return to its baseline state quickly, potentially influencing a more prolonged inflammatory response. This hypothesis is considered because SARS-CoV-2 infection may lead to the development of autoantibodies, which can promote immune dysfunction in the post-COVID state [27]. Furthermore, the persistence of viral antigens and immune complexes may play a role in the immune system’s continuous activation and tissue damage in these individuals [28].

Badinlou and colleagues [29] conducted a longitudinal study investigating self-reported changes in individuals infected with SARS-CoV-2. The authors suggest that the presence of fever may have been reported by participants as a sensation, even in the absence of actual fever, that includes the symptom itself.

Expectoration (Figure 4a) is a symptom that may be related to cough, with the WIMV group also showing the highest intensity (0.63 ± 1.22). Cough can be classified as dry or productive, depending on the presence or absence of sputum [30]. Productive cough is related explicitly to bronchitis or pneumonia caused by infections, such as COVID-19 [31]. However, sputum can occur without a cough, especially in chronic respiratory diseases, such as bronchiectasis or long-term COVID-19, which is also characterized as a chronic condition.

Watase and colleagues [30] reported a correlation between the severity of COVID-19 and the presence of prolonged cough with sputum, suggesting that pneumonia may be associated with these chronic symptoms [32]. This indicates that the severity of the disease is related to prolonged cough and sputum formation [30]. This finding helps explain why the WIMV group (0.63 ± 1.22) presented the highest mean intensity of symptoms, followed by the IMV group (0.61 ± 1.37), as hospitalized patients generally present more severe forms of the disease. Thus, persistent airway inflammation caused by the more severe form of the disease may lead to hyperplasia of the goblet cells, which are responsible for mucus secretion, increasing sputum production in the long term [30].

Regarding Figure 4b, at the beginning of the monitoring period, all groups exhibited relatively higher levels of expectoration, which gradually decreased over the days. Although the WIMV group had the highest mean intensity for expectoration, during the initial days of monitoring, particularly the first two days, the IMV group recorded the highest intensities. This can be explained by managing mechanical ventilation in patients with severe COVID-19, which is consistently correlated with sputum over a 12-month follow-up period [30]. Further, in the study by Watase and colleagues [30], a longitudinal evaluation of cough and sputum production was conducted over the first 12 months following SARS-CoV-2 infection. The patients were assessed at the start of monitoring, at 3, 6, and 12 months, making this the first detailed study on sputum production in post-COVID patients [30]. The authors observed a gradual decrease in the prevalence of sputum over time [30]. This finding is consistent with our results, where we also observed a gradual decrease in the symptoms within our sample, although without complete resolution of the symptom.

The IMV group presented the highest mean intensity for the sore throat symptom (Figure 5a). Upon viral invasion of the body, the immune system begins to release inflammatory mediators in the airways as a response to the infection, causing sore throat [33]. Therefore, as with fever, sore throat is expected to be absent in the absence of an active infection. The low intensities observed across all groups are likely due to the resolution of the infection.

In Figure 5b, we observe that all groups begin with varying levels of sore throat, with the IMV patients presenting the highest initial level. Sore throat tends to decrease rapidly in the first few days for all groups, stabilizing at lower. However, persistent and fluctuating levels, especially in the IMV patients, indicate that these patients may experience a more complicated and prolonged recovery from this symptom. Badinlou and colleagues [29] also identified variations in the prevalence of sore throat throughout their study, where an initial prevalence of 45.4% decreased to 37.0% at three months, increased to 40.9% at six months, and remained at 41.4% at twelve months. A sore throat is considered chronic when it persists for more than 14 days, with non-infectious causes being the most likely, such as physical–chemical factors, including post-intubation [33,34]. This may explain why the IMV individuals exhibited the highest intensities and the most significant fluctuations in intensity during the monitoring period, as they required mechanical ventilation as part of their treatment for COVID-19 during hospitalization.

The WIMV group had the highest mean intensity for the diarrhea symptom. All groups showed similar values with low intensities (Figure 6a). According to the systematic review and meta-analysis by Ghimire and colleagues, patients who experience diarrhea likely have a higher viral load, which may lead to a more intense systemic response and worsen the symptoms [35]. However, since there is no active viral infection among the volunteers in our study, the viral load is not elevated, which justifies the lower intensities observed.

Figure 6b shows that at the beginning of the monitoring period, the WIMV group exhibited the highest levels of diarrhea. Our sample observed considerable variability in all groups, with symptom intensity alternating between high and low peaks continuously. This pattern is similar to that found by Badinlou and colleagues [29], who also identified variations in the prevalence of diarrhea during the longitudinal follow-up of their sample.

Contrary to these findings, Fernández-de-las-Peñas and colleagues [36] evaluated 1266 COVID-19 survivors previously hospitalized in a longitudinal follow-up. In line with the results of the present study, the authors observed a gradual decrease in the prevalence of diarrhea over time [36]. It is known that SARS-CoV-2 can alter the gut microbiome, and these changes have been associated with the severity of the disease [37]. It is suggested that the persistence of gastrointestinal symptoms, such as diarrhea, may be explained by the prolonged presence of the virus in the gut [37]. Hospitalization exposes patients to several factors that promote dysbiosis, such as the use of antibiotics [37,38]. Therefore, the persistence of this symptom in these individuals may be associated with difficulties in restoring a healthy microbiome [37].

Regarding the highest intensities, the symptoms of shortness of breath (Figure 7a), fatigue (Figure 8a), cough (Figure 9a), headache (Figure 10a), body pain (Figure 11a), and other symptoms (Figure 12a) are observed.

The symptom of shortness of breath was most intense in the WIMV group (Figure 7a). Al-Jahdhami and colleagues [39] highlight that shortness of breath was a prominent characteristic in individuals who participated in their study and presented with long-term COVID-19, which is more significant among patients with a more severe form of COVID-19. It includes hospitalized patients, including Intensive Care Units, requiring either invasive or non-invasive oxygen support. This finding is consistent with our results, as the highest symptom intensity was observed in the WIMV group (1.67 ± 2.01), followed by the IMV group (1.47 ± 1.93). It occurs probably due to the severity of the disease in these patients, making complete recovery more challenging.

The inflammatory process during active viral infection leads to the collapse of the alveolar sacs and reduces blood oxygen levels, resulting in hypoxemia [40]. Therefore, persistent inflammation and lung damage contribute to prolonged shortness of breath, especially in those who experienced severe forms of the disease. Throughout the monitoring period, patients in the IMV and WIMV groups exhibited the highest shortness of breath, being highest in the early days of monitoring and gradually reduced over time (Figure 7b). This gradual decrease is consistent with the findings of Grewal and colleagues [41], who conducted a longitudinal study with a prospective cohort of individuals hospitalized due to COVID-19. In the study by Badinlou and colleagues [29], the authors also observed a gradual decrease in the prevalence of shortness of breath over time, although with some variability during the follow-up period. The MG and RG groups maintained consistently low and stable levels.

Fatigue showed the highest mean intensity in the IMV group (Figure 8a). This symptom is one of the most persistent and common among individuals previously infected with SARS-CoV-2, and it is also commonly observed following various viral and bacterial infections [42]. Understanding this symptom’s pathogenesis is still limited, as the cause of fatigue is often unrelated to a single factor [42]. Therefore, according to Rudroff and colleagues [43], post-COVID fatigue can be defined as reduced physical and/or mental performance resulting from central, psychological, and/or peripheral alterations due to COVID-19.

For example, Calabria et al. [44] reported clinically significant fatigue in more than 80% of the participants in their study, with physical fatigue being the most prominent. Furthermore, high levels of apathy, anxiety, and executive dysfunction, as well as difficulties in attention and executive functions, were identified as consistent predictors of the different types of fatigue observed. These findings reinforce the strong association between cognitive neuropsychiatric symptoms and fatigue in patients with post-COVID syndrome.

Ahmad and colleagues [45] and Townsend and colleagues [46] emphasize that they did not find a link between the symptom and disease severity, considering it an independent factor from hospitalization. However, post-COVID individuals may experience more significant impairment due to SARS-CoV-2 infection and develop physiological alterations in the respiratory system, thus increasing the imbalance in their pulmonary structures over time [47].

Regarding the longitudinal monitoring (Figure 8b), the IMV group initially presented the highest intensities of fatigue, followed closely by the WIMV group. The MG and RG groups showed lower symptom intensity from the beginning of the monitoring and remained relatively constant and stable throughout the period. As the days passed, fatigue gradually decreased for all groups, though the IMV and WIMV groups still exhibited the highest intensities throughout the monitoring.

Jacobs and colleagues [48] aimed to identify persistent symptoms of COVID-19 35 days after hospital discharge and assess the impact of these symptoms on patients’ quality of life, health, and physical, mental, and psychosocial functions. In this study, 56.8% of the studied patients reported fatigue at hospital discharge. After 35 days, this symptom was slightly decreased; 55.0% continued to report fatigue. Similarly, in the study of Badinlou and colleagues [29], the prevalence of the symptom also gradually decreased over time. Fernández-de-las-Peñas and colleagues [49] conducted a study to determine fatigue dyspnea levels and long-term symptoms after hospital discharge in COVID-19 survivors. The authors observed no significant differences in fatigue levels between hospitalized individuals and those in more severe conditions who required ICU admission. In our sample, both the WIMV and IMV groups were hospitalized. Therefore, the authors’ findings are consistent with ours, showing that fatigue intensity was similar in both groups, regardless of the severity during hospitalization. Infection with SARS-CoV-2 can cause various persistent symptoms in post-COVID individuals, such as fatigue. However, a more significant number of comorbidities and the presence of more symptoms during the acute phase of the disease may trigger various adverse events, contributing to the persistence of the symptoms in these individuals [49].

One of the most common symptoms after SARS-CoV-2 infection is cough. This symptom is often caused by residual airway inflammation, which leads to hyper-reactivity of these structures. Over time, cough can evolve into a vicious cycle, where excessive irritation and inflammation exacerbate the symptoms [50]. The prevalence of cough among individuals varies significantly in the literature, ranging from 2.1% to 73.6% [51,52]. This variability may explain the different intensities of cough observed in our study (Figure 9a), with prevalence varying across the groups. This can be justified by the predominant involvement of the lower airways and pneumonia, which explains the higher average cough intensity in the WIMV group [50].

Over time, all groups show a trend of decreasing cough intensity (Figure 9b). Our findings are similar to those of Badinlou and colleagues [29], who also observed a gradual decrease in this symptom. Watase and colleagues [30] conducted a longitudinal assessment of cough and sputum production during the first 12 months after SARS-CoV-2 infection. The authors observed a gradually decreasing prevalence of these symptoms [30]. Carfì and colleagues [53] also observed a reduction in the prevalence of cough when comparing the acute phase to the average 60-day follow-up period.

In the most severe cases of COVID-19, such as those that require hospitalization, the progression of pneumonia leads to respiratory failure due to tissue damage, and the entire process of severe pneumonia increases cough sensitivity [30]. Consequently, these individuals may experience pulmonary fibrosis and bronchiectasis [54]. Therefore, due to the disease process, it is speculated that the persistence of cough is a consequence of the sequelae caused by SARS-CoV-2 infection. However, over time, there is a gradual recovery from the damage caused by the disease.

Headache is one of the earliest and most common symptoms during the acute phase of SARS-CoV-2 infection, and it can occur alone or in conjunction with various other symptoms [55]. However, the symptom burden, the characteristics presented, the pathophysiology, the treatment, and its prevalence as a symptom of long COVID are not fully understood [56]. The WIMV group showed the highest average intensity for this symptom (Figure 10a). However, there appears to be no correlation between the severity of the disease and the frequency of headache symptoms for long COVID. Tana et al. [55] and Fernández-de-las-Peñas et al. [57] state that the frequency of headaches in patients with a severe form of the disease is similar to those with a mild form. In line with this hypothesis, Garcia-Azorin et al. [58] conducted a 9-month prospective study evaluating patients with headaches during the acute phase of COVID-19. They observed that the symptom frequency was similar to that of outpatients compared to those who required hospitalization.

Acute headache caused by a systemic viral infection may be associated with a significant systemic immune response [59]. Thus, a hypothesis has been proposed that patients with prolonged headaches due to COVID-19 may manifest a persistent activation of the immune system, which could be correlated with the enduring impact of this symptom in individuals in the present study [55]. However, evidence of the immune response over time in patients with prolonged headaches remains limited [55].

At the start of the monitoring period, all groups presented similar levels for the intensity of headache (Figure 10b). A sharp decrease was observed on the second day for the IMV group and marked fluctuations throughout the remainder of the monitoring period. The WIMV group maintained higher levels of headache intensity over time, while the MG and RG groups showed less pronounced variations. Despite these fluctuations, headache intensity tends to decrease in all groups over time, though with numerous fluctuations over the 15 days. In the study by Badinlou et al. [29], a sharp decline in the prevalence of headaches was observed, although there was some stabilization in the following months. Fernández-de-las-Peñas et al. [57] conducted a meta-analysis to investigate headache’s temporal course and differentiate between hospitalized and non-hospitalized patients. The authors reported results similar to ours, showing variation in symptom prevalence over time but with a tendency for a reduction in prevalence by the end of the follow-up period [57]. They also observed no significant difference in the prevalence of post-COVID headaches between hospitalized and non-hospitalized patients during follow-up periods [57]. The hypotheses regarding headache pathogenesis include viral persistence, microthrombi, or even continuous immune system activation [60].

Muscle pain in long COVID can be considered a multifactorial symptom, where the relationship between SARS-CoV-2 infection is associated with the host’s immune response, external factors during the active phase of the disease, such as hospitalization, and emotional factors arising during the disease outbreak [61]. According to Fernández-de-las-Peñas et al. [61], the prevalence of musculoskeletal pain in the post-COVID phase was nearly 80%. Neto et al. [62] report that in their study, patients hospitalized due to COVID-19 were more likely to experience late musculoskeletal pain than non-hospitalized patients. These individuals also exhibited the worst disability scores and higher levels of pain. Those who reported pain also had a more severe clinical course during hospitalization [62]. In close agreement with the results described in Figure 11a, the authors identified a positive correlation between the duration of IMV and pain intensity, indicating that individuals who underwent prolonged IMV had greater pain intensity [62].

In the longitudinal monitoring of body pain (Figure 11b), the IMV group exhibited the highest levels of pain, which gradually decreased over the monitoring period, though remaining elevated. Individuals in the WIMV group presented elevated and stable levels of body pain. Meanwhile, the MG and RG groups showed the lowest intensities, which remained stable throughout the monitoring period.

The study of Badinlou et al. [29] divided body pain into two categories: pain in various parts of the body and joint pain. Both types of pain showed a tendency for gradual reduction over time. Steinmetz et al. [63] conducted a prospective cohort study. They observed increased joint pain and myalgia during the acute/initial phase, which only started to decrease after three months, reducing further after six months [63]. Both pain-related symptoms tended to decrease over time.

Fernández-de-las-Peñas et al. [64] conducted a study investigating the prevalence of musculoskeletal pain post-COVID in hospitalized individuals. The authors highlighted that the prevalence of the symptom was 43.4% after 8.4 months post-hospital discharge, decreasing to 37.8% after 13.2 months, indicating a gradual decrease in the symptom’s prevalence over time [64]. Karaarslan et al. [65] conducted a short-term follow-up of hospitalized COVID-19 patients. The authors observed that the prevalence of musculoskeletal symptoms was 92.3% during hospitalization, decreasing to 72.7% after 14 days post-discharge and to 56.3% after one month, showing a gradual decrease in symptom prevalence over time [65].

In our sample, the IMV group exhibited the highest intensities of body pain, followed by the WIMV group. These results are in agreement with previous studies suggesting that individuals requiring hospitalization have a higher likelihood of developing delayed body pain [62]. This may occur through indirect mechanisms such as muscle atrophy and neuropathy associated with prolonged immobilization due to hospitalization. In addition, it may also be associated with the use of IMV and neuromuscular blocking drugs, painful neurological complications of the disease, and psychological factors such as post-traumatic stress associated with ICU stays. Also, in line with these propositions, the patients who exhibited the highest levels of body pain in our sample were hospitalized and required IMV (Figure 11a).

Regarding other symptoms, the IMV group again showed the highest average (Figure 12a) and most persistent intensities (Figure 12b). Although various fluctuations occurred throughout the monitoring period, there was a general trend of gradual reduction in intensities over time.

As observed in Table 4, among the other symptoms, the most prevalent was memory lapse (10.5%). In line with this finding, Teodoro et al. [66] highlight that cognitive difficulties, such as memory lapses and lack of concentration, are among the most common neurological manifestations post-COVID. Steinmetz et al. [63] observed that the frequency of memory difficulties during the acute phase of COVID-19 infection was 25.7%. This frequency decreased to 19.5% at the start of the study, and after three months, it saw a more pronounced reduction to 2.7% [63]. However, at six months, the frequency increased again to 10.0% [63]. Furthermore, Guo et al. [67] identified a consistent pattern of memory deficits in patients who had COVID-19, correlating these deficits with the severity of the reported symptoms. It is important to highlight that patients who require endotracheal intubation during hospitalization for COVID-19 are more likely to develop neuropsychiatric symptoms and musculoskeletal involvement, meaning they are patients with a higher tendency to present sequelae [68].

As described in Figure 13 and detailed in Table A1, the symptom with the highest intensity was fatigue (34.44 ± 35.99), followed by body pain (24.24 ± 30.67) and shortness of breath (19.63 ± 29.73). The literature supports this pattern. Taylor et al. [69] identified fatigue as the most common symptom in their study, followed by musculoskeletal pain. Similarly, Mandal et al. [70] found that fatigue was the most frequent symptom, followed by shortness of breath. Irisson-Mora et al. [68] also conducted a study with patients discharged after recovering from COVID-19. They showed that the most prevalent symptoms in the general population were fatigue and shortness of breath.

A 2021 meta-analysis also highlighted fatigue and dyspnea as the most prevalent post-COVID symptoms [71]. Tana et al. [55] suggested that fatigue, shortness of breath, body pain, and cough were the four most common symptoms, followed by headache. This finding is consistent with our research results, where headache was identified as the fourth most intense symptom, following fatigue, body pain, and shortness of breath.

Figure 14 and Table A2 show that the IMV and WIMV groups presented the highest average sum of symptoms. These findings are also consistent with the literature. The meta-analysis by Yuan et al. [72] and the cohort study by Pérez-González et al. [73] showed that hospitalized patients frequently had more symptoms than those not hospitalized. These findings can be explained by the severity of the disease, as addressed in a systematic review by Maglietta et al. [74], which discussed how disease severity is a risk factor for developing one or more symptoms after COVID-19 recovery. Pérez-González et al. [73] also stated that symptom persistence was directly related to the severity of COVID-19. These authors compared ICU patients, who were more severely ill, with those hospitalized in general wards and had less severe symptoms but found no association between these groups and symptom persistence [73]. This confirms our findings, where the IMV and WIMV groups showed similar averages with no statistically significant differences.

Figure 15 presents the longitudinal follow-up analysis of the symptoms’ sum. The IMV group shows the highest intensity yet is similar to the WIMV group. The intensity levels between these groups remained similar throughout the monitoring period, showing a gradual decrease. Sudre et al. [75] reported similar data, where the prevalence of symptoms gradually decreased over time.

Unlike those with milder forms of the disease, individuals who require hospitalization are more susceptible to developing long-term symptoms [76]. This can be attributed to mechanical ventilation and/or prolonged immobilization during hospitalization rather than the disease [76]. Therefore, these individuals are expected to take longer to recover [76]. This may explain why, in our analysis, the IMV and WIMV groups exhibited higher symptom intensities during the longitudinal follow-up.

In Figure 16, we observed that the MG group presented higher values for both SBP and DBP, indicating a higher prevalence of hypertension in the MG group. However, these values remain within normal limits. Fedorowski et al. reported that Cardiovascular Autonomic Dysfunction (CAD) is an important component of post-COVID, potentially affecting at least one-third of symptomatic COVID-19 patients [77]. CAD occurs when the cardiovascular system does not function properly due to disordered homeostatic control by the autonomic nervous system, encompassing a variety of conditions rather than a single specific disorder. Among the conditions of CAD, we can highlight hypertension as one of the most typical findings observed in the post-COVID phase [77].

In several individuals, symptoms suggestive of CAD may have been present prior to COVID-19 and may be exacerbated after infection with SARS-CoV-2. In other cases, CAD can develop after the infection, showing a direct temporal association between COVID-19 and the onset of CAD [77]. However, Choutka et al. [78] observed that CAD is unrelated to the severity of COVID-19 and can arise after mild, moderate, or even asymptomatic forms of the disease. Daugherty et al. [79] also mentioned that, in addition to typical clinical sequelae, the risk of developing hypertension is nearly twice as high after infection with SARS-CoV-2 compared to the general population. Evidence is still insufficient to establish hypertension as a risk factor for long-term COVID-19. However, Tleyjeh et al. [80] conducted an observational study that indicated pre-existing hypertension as a predictor for long-term COVID-19.

Our study has limitations that should be emphasized. The COVID-19 vaccination process, the resumption of daily activities, and the end of the pandemic led to less available time for the necessary management of home monitoring, making recruiting volunteers more difficult due to the reduction in people’s time availability.

Secondly, in individuals from the IMV group, we observed anxiety and some discomfort when discussing the course of COVID-19, which hindered the recruitment of volunteers willing to participate in our study within this group.

Thirdly, a relevant limitation of this study is the temporal heterogeneity of the sample. Participants were enrolled at different points during the COVID-19 pandemic, which means they were exposed to different SARS-CoV-2 variants. This diversity may have influenced the symptoms presented and the clinical progression of the patients. However, we believe that this heterogeneity also reflects the real-world context of the population affected by post-COVID syndrome, providing greater practical applicability of the study across varied scenarios.

It could be argued that one of the four studied groups (IMV) does not reach the estimated value for the number of patients. Future studies should include a larger number of subjects. However, this preliminary analysis significantly contributes to an important debate in the literature concerning the symptoms in COVID-19 patients recovering from Invasive Mechanical Ventilation.

Another limitation is the lack of correlation between the findings and functional tests. These analyses could have provided a more specific assessment of pulmonary changes associated with the intensity of respiratory symptoms presented, as well as the impact on the daily living activities of these individuals.

Finally, blood pressure was investigated in only two classes of patients. This analysis was included after the start of symptom monitoring and the observation of many patients reporting arrhythmias. The presented results can be considered a pilot study, allowing for a preliminary assessment. Further investigations involving a more significant number of subjects are warranted.

## 5. Conclusions

In conclusion, post-COVID-19 home monitoring of symptoms is a crucial component of comprehensive care, facilitating early detection of complications, tracking long-term effects, reducing the burden on healthcare systems, personalizing care, and promoting patient engagement. It represents a shift toward proactive and patient-centered healthcare after the pandemic.

Patients in the WIMV group exhibited the highest average intensity in symptoms such as fever, sputum production, diarrhea, cough, shortness of breath, and headache. In contrast, the IMV group had the highest average intensity regarding sore throat, fatigue, body pain, and other symptoms. In general, the symptom with the highest intensity was fatigue, followed by body pain and shortness of breath. Memory lapses were another prevalent symptom among the monitored individuals.

Despite fluctuations in symptom intensity over time, there was a general trend of gradual reduction in most symptoms. However, hospitalized patients exhibited higher levels and greater prevalence of symptoms. Blood pressure was higher in the MG group than in the GR group, but the values remained within the normal range.

These results provide novel insights, revealing distinct differences in the symptom profiles among the studied groups. These findings have significant implications for developing personalized care strategies and informing future pandemic preparedness and response efforts.

## Figures and Tables

**Table 1:** Mean ± SD of the anthropometric characteristics of the studied groups.

	MG (A, n = 40)	WIMV (B, n = 40)	IMV (C, n =13)	RG (D, n = 40)	*p*
Age (years)	43.9 ± 15.5	52.8 ± 11.2	52.1 ± 12.2	40.4 ± 12.4	A-B,C-D,A,C,B-D
Height (cm)	164.9 ± 7.4	167.5 ± 10.6	164.9 ± 12.0	164.2 ± 8.8	A,B,C,D,A,C,B,D
Weight (kg)	76.3 ± 16.1	83.8 ± 15.7	92.5 ± 22.2	74.9 ± 14.0	A-B,C-D,A-C,B-D
BMI (kg/m^2^)	28.1 ± 6.1	29.9 ± 5.2	33.9 ± 6.2	27.8 ± 4.6	A,B-C-D,A-C-B,D
Gender (F/M)	(29/11)	(21/19)	(7/6)	(29/11)	-

Abbreviations: MG—Mild Group; WIMV— Post-Discharge Without Invasive Mechanical Ventilation; IMV— Post-Discharge With Invasive Mechanical Ventilation; RG—Reinfected Group; BMI—body mass index; n = number of patients. Notes: “-” indicates a significant difference, while “,” represents non-significant differences.

**Table 2:** Clinical characteristics of the studied patients.

	MG n (%)	WIMV n (%)	IMV n (%)	RG n (%)
Current smoker	1 (2.5)	0 (0)	1 (7.7)	2 (5.0)
Former smoker	4 (10.0)	13 (32.5)	2 (15.4)	3 (7.5)
Overweight	15 (37.5)	15 (37.5)	1 (7.7)	14 (35.0)
Obesity	10 (25.0)	15 (37.5)	8 (61.5)	14 (35.0)
Severe obesity (BMI ≥ 40.0)	2 (5.0)	3 (7.5)	3 (23.1)	0 (0)
Diabetes mellitus	1 (2.5)	10 (25.0)	4 (30.7)	2 (5.0)
Prediabetes	2 (5.0)	2 (5.0)	0 (0)	0 (0)
Arterial hypertension	8 (20.0)	17 (42.5)	8 (61.5)	6 (15.0)
Cardiomegaly	1 (2.5)	0 (0)	1 (7.69)	0 (0)
Hypercholesterolemia	3 (7.5)	0 (0)	0 (0)	0 (0)
COPD (pulmonary emphysema and chronic bronchitis)	0 (0)	1 (2.5)	0 (0)	1 (2.5)
Asthma	2 (5.0)	4 (10.0)	0 (0)	3 (7.5)
Home O_2_ dependence	0 (0)	0 (0)	1 (7.7)	0 (0)
Anxiety	0 (0)	0 (0)	0 (0)	4 (10.0)
Depression	0 (0)	1 (2.5)	1 (7.7)	1 (2.5)
Used Hydroxychloroquine	0 (0)	2 (5.0)	1 (7.7)	1 (2.5)
Used Azithromycin	11 (27.5)	18 (45.0)	7 (53.8)	13 (32.5)
Used Ivermectin	4 (10.0)	9 (22.5)	3 (23.1)	3 (7.5)
Hospital stay (days)	-	18.1 ± 16.0	49.8 ± 26.6	-

Abbreviations: MG—Mild Group; WIMV—Post-Discharge Group Without Invasive Mechanical Ventilation; IMV—Post-Discharge Group With Invasive Mechanical Ventilation; RG—Reinfected Group; n—number of patients.

**Table 3:** Mean ± SD of the intensity of monitored symptoms.

	MG	WIMV	IMV	RG
Shortness of breath	1.12 ± 2.26	1.67 ± 2.01	1.47 ± 1.93	1.07 ± 2.12
Fatigue	1.72 ± 2.61	2.92 ± 2.60	3.28 ± 2.31	1.92 ± 2.62
Fever	0.15 ± 0.57	0.30 ± 0.80	0.16 ± 0.57	0.11 ± 0.54
Cough	0.98 ± 1.75	1.54 ± 1.90	1.08 ± 1.65	0.62 ± 1.50
Expectoration	0.48 ± 1.13	0.63 ± 1.22	0.61 ± 1.37	0.58 ± 1.58
Headache	1.18 ± 2.44	1.40 ± 2.00	0.89 ± 1.92	1.25 ± 2.37
Sore throat	0.29 ± 0.89	0.41 ± 0.91	0.43 ± 1.20	0.21 ± 0.87
Body pain	1.16 ± 1.22	2.01 ± 2.32	2.77 ± 2.43	1.29 ± 2.19
Diarrhea	0.19 ± 0.87	0.40 ± 1.06	0.14 ± 0.61	0.16 ± 0.70
Other symptoms	1.41 ± 2.44	1.34 ± 2.07	1.83 ± 2.21	0.70 ± 2.02

Abbreviations: MG—Mild Group; WIMV—Post-Discharge Group Without Invasive Mechanical Ventilation; IMV—Post-Discharge Group With Invasive Mechanical Ventilation; RG—Reinfected Group.

**Table 4:** Other symptoms reported by the volunteers included in the study.

	n (%)
Memory lapse	14 (10.5)
Tachycardia	7 (5.26)
Tingling in limbs	4 (3.0)
Radiating pain	3 (2.25)
Anxiety	3 (2.25)
Chest pressure	3 (2.25)
Sensitive smell	2 (1.50)
Weakness	2 (1.50)
Arterial hypertension	2 (1.50)
Anosmia	2 (1.50)
Hair loss	2 (1.50)
Dizziness	2 (1.50)
Nasal congestion and runny nose	2 (1.50)
Sneezing	1 (0.75)
Tremors in limbs	1 (0.75)
Eye pain	1 (0.75)
Ear blockage	1 (0.75)
Testicular inflammation	1 (0.75)
Wart	1 (0.75)
Colic	1 (0.75)
Reflux	1 (0.75)
Nausea	1 (0.75)
Insomnia	1 (0.75)
No other symptoms	75 (56.4)

## Data Availability

The dataset supporting the conclusions of this article will be available on request in the Open Science Framework repository at the following link: https://osf.io/5tvgu/ (accessed on 20 April 2025).

## Abbreviations

The following abbreviations are used in this manuscript:

BMI	Body mass index
BP	Blood pressure
CEP/HUPE	Research Ethics Committee of Pedro Ernesto University Hospital
COVID-19	Coronavirus Disease 2019
DBP	Diastolic Blood Pressure
ICU	Intensive Care Unit
IMV	With Invasive Mechanical Ventilation group
MG	Mild Group
n	Number of patients
NIV	Non-invasive ventilation
ns	Not significant
RG	Reinfected Group
RPM	Remote patient monitoring
SARS-CoV-2	Severe acute respiratory syndrome coronavirus 2
SBP	Systolic Blood Pressure
SD	Standard deviation
WIMV	Without Invasive Mechanical Ventilation group

## Appendix A

### Appendix A.1

**Table A1:** Mean ± SD of the intensity of each symptom in all volunteers.

Fatigue	34.44 ± 35.99
Body pain	24.24 ± 30.67
Shortness of breath	19.63 ± 29.73
Headache	18.66 ± 28.07
Other symptoms	18.28 ± 29.62
Cough	15.77 ± 21.85
Expectoration	8.56 ± 17.11
Sore throat	4.83 ± 10.28
Diarrhea	3.63 ± 8.79
Fever	2.80 ± 6.82

### Appendix A.2

**Table A2:** Mean ± SD of the intensity of all symptoms presented by each.

IMV	190.61 ± 129.34
WIMV	190.10 ± 134.48
MG	130.42 ± 189.37
RG	119.17 ± 146.91

Abbreviations: MG—Mild Group; WIMV—Post-Discharge Group Without Invasive Mechanical Ventilation; IMV—Post-Discharge Group With Invasive Mechanical Ventilation; RG—Reinfected Group.
